# Challenges of using generative AI for patient education in chronic heart failure: an evaluation of content quality, readability, and actionability in cross-platform LLM-generated texts

**DOI:** 10.3389/fpubh.2026.1801829

**Published:** 2026-03-05

**Authors:** Zhiqiang Wang, Xiaoya Li, Chao Ma, Zhiwen Zhang

**Affiliations:** 1 Yangtze University Medical School, Jingzhou, Hubei, China; 2 Department of Cardiology, Fuwai Central China Cardiovascular Hospital, Central China Fuwai Hospital of Zhengzhou University, Zhengzhou, Henan, China

**Keywords:** actionability, chronic heart failure, cross-platform evaluation, information quality, LLMs, patient safety, readability, self-management education

## Abstract

**Objective:**

To compare the differences in content quality, readability, and actionability of patient education texts for self-management of chronic heart failure (CHF) generated by five mainstream large language models (LLMs) in China, and to provide a basis for platform selection and assessment framework construction for clinical use.

**Methods:**

A standardized set of 20 questions was developed based on literature review, guidelines, and consensus from cardiovascular experts, covering disease awareness, diagnosis and classification, treatment and rehabilitation, daily management and prevention, and psychosocial dimensions. Using a uniform prompt, responses were generated by DeepSeek-R1, Doubao, ERNIEBot 4.5 Turbo, Qwen3-Max-Thinking-Preview, and Kimi K2. The PEMAT-P scale was used to assess understandability and actionability, 36-item expanded EQIP (EQIP-36 score) scale was used to evaluate information completeness and standardization, and Global Quality Score (GQS) was used to assess overall quality. Additionally, seven readability formulas, including Flesch Reading Ease Score (FRES) and Flesch–Kincaid Grade Level (FKGL), were computed for comparison.

**Results:**

Overall quality was high [GQS median 5.00 (4.00–5.00)] with significant between-platform differences (χ^2^ = 14.47, *P* = 0.006). Doubao and Kimi K2 achieved the highest GQS [both 5.00 (5.00–5.00)]. DeepSeek-R1 showed the greatest information completeness [EQIP-36 39.20 (36.17–44.23); χ^2^ = 25.07, *P* < 0.001] but the lowest readability [FRES 19.32 (17.94–36.89) and FKGL 14.28 (13.02–15.85); both *P* < 0.001]. ERNIEBot 4.5 Turbo and Qwen3-Max-Thinking-Preview were most readable (FRES ≈ 59; FKGL ≈ 8; both *P* < 0.001) but had lower EQIP-36 scores. Actionability was limited overall [PEMAT-P actionability 20.00% (0.00–40.00); χ^2^ = 26.40, *P* < 0.001] and varied by topic, with daily management and prevention outperforming disease knowledge and diagnosis/classification (χ^2^ = 20.86, *P* < 0.001).

**Conclusion:**

LLMs show potential for use in patient education for CHF, but there is a structural trade-off between information detail and readability, as well as gaps in actionability and verifiability. It is recommended to combine enhanced search and structured template generation strategies, and establish a governance feedback loop involving prompt engineering, clinical expert review, and continuous monitoring to improve readability alignment, completeness of action instructions, and patient safety.

## Introduction

Chronic heart failure (CHF) is a chronic illness with high burden, and its incidence, mortality risk, and healthcare utilization impose a substantial population-level burden (1–3). It is approximated that about 64 million individuals worldwide experience heart failure (4). Despite advances in treatment, patients remain at high risk of adverse outcomes, with nearly half dying within 5 years after the initial diagnosis (4). Readmissions are common, with approximately one quarter of patients readmitted within 30 days after discharge and about half within 6 months (5–10). Repeat readmissions increase healthcare utilization and are associated with poorer prognosis and lower quality of life (5–10). Accordingly, self-management and adherence to recommended treatment are central to long-term CHF care (11–13). Contemporary guidelines emphasize patient education and self-management support to improve symptoms and reduce readmission and mortality risk (14). The ACC/AHA heart failure guideline highlights multidisciplinary self-care education, including medication management, diet and exercise, and symptom monitoring, to reduce readmissions and improve survival (14).

The implementation of these strategies is often constrained by the quality, readability, and practical usability of patient education materials. Prior studies indicate that many health education resources exceed the health literacy level of the general population (15, 16). The American Medical Association (AMA) and National Institutes of Health (NIH) recommend that patient education resources be written at a 5th−6th grade reading level to improve accessibility (17). However, heart failure education materials are frequently written at high school or college level (15), and online CHF resources often have a median reading level of 9th−12th grade or higher, with few meeting recommended thresholds (15). Excessive reading difficulty may reduce comprehension and adherence, particularly among individuals with limited health literacy, and systematic reviews and meta-analyses have linked low health literacy in heart failure populations to higher mortality and readmission risk (18). Beyond readability, educational materials may lack explicit action steps that translate information into behavior. PEMAT-based evaluations indicate that materials with good understandability do not necessarily achieve adequate actionability, often below commonly used thresholds such as 60% (19). Limited actionable guidance can weaken the pathway from knowledge acquisition to self-management behaviors, potentially diminishing the effectiveness of educational interventions (19).

With advances in generative AI, large language models (LLMs) have entered health information delivery and patient education, enabling scalable and potentially customizable educational content (20). Past studies indicate that more advanced models bear a limited ability to modify tone and easier reading (21). LLMs have evolved from general-purpose pretrained models to instruction-following conversational systems through alignment and instruction-tuning approaches, improving fluency and controllability in user-facing interactions. Recent deployments increasingly support longer-context dialogue and, in some settings, retrieval- or tool-augmented generation to enhance coverage and reduce unsupported claims. These developments strengthen the potential of LLMs for patient education, but they also introduce persistent challenges, including variability across prompts and model updates, uncertain medical accuracy, and limited citation and verifiability, which may increase misinformation risk and hinder professional review (22). Comparative studies further suggest that different chatbots can vary substantially in textual complexity and informational density for similar medical topics (23). In CHF, systematic cross-model comparisons of LLM-generated self-management education that simultaneously assess content quality, readability, and actionability remain limited. To address this gap, we compared five widely used Chinese LLM platforms [DeepSeek-R1 (DeepSeek, Hangzhou, Zhejiang, China); Doubao (ByteDance Ltd., Beijing, China); ERNIEBot 4.5 Turbo (Baidu, Beijing, China); Qwen3-Max-Thinking-Preview (Alibaba Cloud, Hangzhou, Zhejiang, China); Kimi K2 (Moonshot AI, Beijing, China)] using standardized prompts and scenario settings to generate CHF self-management education texts. Content quality was evaluated using PEMAT-P (understandability and actionability), EQIP-36 (information completeness and standardization), and GQS (overall quality). Readability was assessed using seven metrics (FKGL, FRES, GFOG, CLI, SMOG, ARI, and LW). This study provides comparative evidence to inform platform selection in clinical communication and offers a structured methodological reference for evaluating LLM-generated patient education materials in healthcare.

## Methods

### Study design and question collection

This cross-sectional observational study aimed to identify common public concerns about chronic heart failure (CHF) and to develop a systematic, clinically relevant, and representative question bank for evaluating the performance of large language models (LLMs) in generating patient-facing health information. It also seeks to mark out the areas of focus of the populace, as a measure of refining patient education and management. To this end, the study first prepared a question bank of 20 common questions based on an extensive study of the literature and other authoritarian guidelines, and was completed by consulting the domain experts (Table 1). These questions are based on five major dimensions that include disease basic understanding, diagnosis and classification, treatment and rehabilitation, daily management and prevention, and psychosocial factors and prognosis. These questions were designed in line with other similar studies in the past to guarantee clinical feasibility (24, 25). Seeking the authority and representativeness of all questions, the integrated list was first reviewed by three cardiovascular experts, all with more than 10 years of experience, and validated by them, which in turn became finalized by a consensus process. The present systematic multi-step design has provided this research with a carefully selected list of questions that provide a valuable basis of further quantifying the quality of the answers generated by AIs, their readability, usability, and reliability.

**Table 1:** List of common questions regarding chronic heart failure (CHF).

Issue list
**Basic understanding of the disease**
1. What type of disease is chronic heart failure?
2. What is the main pathogenesis of chronic heart failure?
3. What is the most common etiology of chronic heart failure?
4. Does chronic heart failure have a genetic predisposition?
**Diagnosis and classification**
1. What is the most commonly used biomarker for diagnosing chronic heart failure?
2. What is the preferred imaging examination for confirming chronic heart failure?
3. How is chronic heart failure clinically classified according to left ventricular ejection fraction?
4. How is chronic heart failure functionally graded according to New York Heart Association (NYHA)?
**Treatment and rehabilitation**
1. What are the core drug categories for the basic treatment of chronic heart failure with reduced ejection fraction (HFrEF)?
2. What is the main role of SGLT2 inhibitors in the treatment of chronic heart failure?
3. Under what circumstances should implantation of cardiac resynchronization therapy (CRT) be considered for chronic heart failure patients?
4. What are the stable conditions for chronic heart failure patients to start cardiac rehabilitation training?
**Daily management and prevention**
1. What component needs to be most restricted in the dietary management of chronic heart failure patients?
2. What are the basic principles of fluid intake management for chronic heart failure patients?
3. What is the purpose of daily weight monitoring for chronic heart failure patients?
4. What signs in chronic heart failure patients indicate a possible acute exacerbation?
**Psychosocial factors and prognosis**
1. What is the most common psychological issue among chronic heart failure patients?
2. What psychological adjustment methods are commonly used by chronic heart failure patients?
3. Does chronic heart failure affect the work capacity of patients?
4. Under what circumstances should palliative care assessment be initiated for chronic heart failure patients?

### AI platform-based question-and-answering and data collection

This study selected five commonly used conversational AI platforms in China, each equipped with a large language model, as the subjects for research. These platforms include DeepSeek-R1, Doubao, ERNIEBot 4.5 Turbo, Qwen3-Max-Thinking-Preview, and Kimi K2.

DeepSeek-R1, released on January 20, 2025 by DeepSeek, is a reasoning-oriented large language model that is open-sourced under the MIT License and is reported to achieve performance comparable to OpenAI's o1 on math, coding, and multi-step reasoning tasks. Doubao is ByteDance's flagship AI assistant product; the service is operated by Beijing Chuntian Zhiyun Technology Co., Ltd., and the Doubao (Seed) large-model family was officially released in May 2024 via Volcano Engine. Publicly reported adoption metrics indicate rapid scaling, with the Doubao app surpassing 157 million monthly active users in August 2025 and Volcano Engine reporting average daily token usage on the order of 50 trillion. Baidu released ERNIEBot 4.5 Turbo on April 25, 2025 as a faster, lower-cost variant of ERNIE 4.5 with multimodal capability and an emphasis on practical developer-facing deployment. Alibaba Cloud's Tongyi Qianwen team announced an early preview of the Qwen3-Max-Thinking reasoning model on November 3, 2025, positioning it as a >1T-parameter reasoning-enhanced model and highlighting test-time scaling and tool-call–assisted reasoning. Moonshot AI open-sourced Kimi K2 on July 11, 2025; it is a trillion-parameter mixture-of-experts model with sparse activation (32B activated parameters) and is positioned for coding and agentic tool use.

All questions were put in English so as to be consistent and comparable in the evaluation process. With every pre-designed question, a new and independent session on each platform was launched. We cleared down previous conversation history and model cache before every question, just to clear any residue of a previous conversation. It is a process that followed the standard evaluation procedures as laid down in current research and it is important to note that every question was asked in a new session and with no prior experience of history so that there would be independent and comparable responses that would be produced on various media. With regards to the interaction strategy, to ensure that the evaluation method in itself does not interfere with the output, a single-round, non-iterative questioning technique was chosen. Each question was only fed one response to the platform and the initial reply obtained on each question was noted verbatim, with no follow-up questions, rephrasings or multi-round interchange. All the tests were done on the same date, December 22, 2025. The preset questions of the 20 questions were entered sequentially into all platforms and the basic information of the generated answers such as the total number of words and the number of sentences was recorded to the letter. The AI responses were stored in raw format, with no human-involvement and editing. The subsequent in-depth and objective evaluation and analysis will be done by using these initial output data as the main material.

### Evaluation tools and indicators

In an attempt to analyze the performances of five LLMs, DeepSeek-R1, Doubao, and ERNIEBot 4.5 Turbo, Qwen3-Max-Thinking-Preview, and Kimi K2, in a more thorough manner, we used three sets of assessment instruments and pointers, each paying attention to various aspects of the quality of medical information. The selection of these instruments was theory- and use-case–driven: CHF self-management education requires not only overall perceived quality, but also clear, actionable instructions and complete, standardized information elements. Therefore, we combined complementary tools to capture (1) patient-oriented understandability and actionability, (2) information completeness and standardization, and (3) global quality appraisal, which together provide a structured, multi-dimensional evaluation aligned with the study objectives. All responses generated by AI used were organized in a similar manner following an export, IDs of platform/models were removed, and random numbers were assigned to reduce the possible bias of the evaluators due to the prior acquaintance of the origin. Two researchers having the experience in clinical and health communication assessment independently conducted the evaluations and applied the PEMAT-P, EQIP-36 score, and GQS scoring systems. Specifically, PEMAT-P was used to quantify patient-facing communication quality by assessing understandability and actionability, which directly reflect whether a reader can comprehend the content and identify concrete self-management steps. EQIP-36 was applied to evaluate whether key informational components are presented in a complete and standardized manner, addressing content structure and completeness beyond readability alone. GQS provided a global, user-centered summary rating of overall quality and perceived usefulness, complementing the more granular checklist-based tools. They were pre-trained on standardized assessments and tested on a pre-selected sample before scoring so that the two evaluators can equip the scoring standards. In instances where disparities between evaluators occurred, deliberations were made, to settle on an agreement. In case of the failure to reach a consensus a senior professional who has had experience in handling heart failure acted as a tiebreaker. The scoring was done, and the consistency of the two evaluators was determined quantitatively with the coefficient of kappa of Cohen. A Kappa is nearer to 1 implying high inter-rater agreement. The Cohens kappa coefficient was 0.88 which implies that there was high inter-rater agreement. In addition, we quantified linguistic difficulty using multiple readability indices to provide an objective characterization of text complexity; this was necessary because actionability and information completeness can trade off against readability, and a single metric cannot capture these competing properties. Such tools evaluate the levels of content comprehension, the general information credibility and reading the text as explained in the subsections below.

#### Understandability assessment

The Patient Education Materials Assessment Tool for Print (PEMAT-P) is used to measure the understandability and actionability of materials. PEMAT-P includes a series of items scored using a binary yes/no approach, which is then converted into a percentage score (26). A higher PEMAT-P score indicates that the text is more accessible and easier for readers to act upon (26).

#### Quality assessment

The EQIP-36 score is a patient information quality scale comprising of 36 evaluation items. This tool evaluates the completeness, accuracy and standardization of the health information and concentrates on the content of the information, source of information description and formatting. The aggregate score represents the quality of the information as a whole (27). The quality of health information on the internet has been determined using the EQIP-36 score which has proven to be reliable and valid in similar research work (28).

Global Quality Score (GQS) is a subjective rating scale to assess the overall usefulness and reliability of the individual response, the scale has a range between 1 (very poor quality) to 5 (very high quality) (29).

#### Readability indicators

Readability indicators of seven classic English readability formulas were subsequently calculated through each response: ARI, CLI, FKGL, FRES, GFOG, LW and SMOG (15, 30–34). These signs give an approximation of the reading difficulty longitudinarily by investigating word and sentence structure of the content. There is an equivalent scoring range and meaning in each indicator. All six indicators are related to U. S. educational grade levels, except FRES, which have lower scores that signify lower educational levels needed and easier reading instructions. FRES on the contrary scores between 0 and 100 such that the higher the score, the more comprehensible the text becomes. These readability indicators were computed with special software to control uniformity in the appraisal. Readability analysis helps objectively compare the complexity of language across different platform responses, and, when combined with subjective ratings, assesses whether the content is suitable for general public reading comprehension.

### Statistical methods

All continuous variables were first assessed for distribution using the Shapiro–Wilk normality test. Since most variables were non-normally distributed, non-parametric methods were applied as the primary analytical approach. Continuous data are expressed as median (Q_1_, Q_3_). For comparisons across LLM platforms (five groups) or topic dimensions (five groups), the Kruskal–Wallis *H* test was used, and the corresponding test statistic is reported as χ^2^ with two-tailed *P* values. When overall differences were significant, *post hoc* pairwise comparisons were conducted using Dunn's test with Bonferroni correction. For comparisons between two groups, the Mann–Whitney *U* test was applied. Effect sizes were reported as *r* (*r* = *Z*/√*N*) for two-group comparisons and epsilon-squared (ε^2^) for Kruskal–Wallis tests. Inter-rater agreement was assessed using Cohen's kappa coefficient. Associations between variables were examined using Spearman's rank correlation. All tests were two-tailed, with statistical significance set at *P* < 0.05. All statistical analyses were performed using IBM SPSS Statistics v25 (IBM Corp., Armonk, NY, USA), and figures were generated using GraphPad Prism v9 (GraphPad Software, San Diego, CA, USA).

## Results

### Text characteristics

#### Characteristics of chronic heart failure educational texts generated by different LLMs

Among 100 CHF educational texts, the median GQS was 5.00 (4.00–5.00), with significant platform differences (Kruskal–Wallis χ^2^ = 14.47, *P* = 0.006). Doubao [5.00 (5.00–5.00)] and Kimi K2 [5.00 (5.00–5.00)] had higher scores, while DeepSeek-R1 scored lower [4.50 (4.00–5.00)]. There were also significant differences in text length and structure (Words: χ^2^ = 55.19, *P* < 0.001; Sentences: χ^2^ = 51.39, *P* < 0.001): the median word count was 285.00 (226.00–363.00), with DeepSeek-R1 having the longest text at 530.00 (418.50–565.50) words and Qwen3-Max-Thinking-Preview the shortest at 201.50 (179.75–228.75). The median number of sentences was 21.00 (15.75–28.00), with DeepSeek-R1 having the most at 37.00 (24.50–39.50) and Kimi K2 the fewest at 14.00 (11.75–15.00). The PEMAT-P score, expressed as a percentage, showed a median understandability score of 69.23% (69.20%−76.90%; χ^2^ = 20.89, *P* < 0.001); the median actionability score was only 20.00% (0.00%−40.00%; χ^2^ = 26.40, *P* < 0.001), with Doubao having the lowest score of 0.00% (0.00%−5.00%), indicating significant differences between platforms in their ability to translate information into actionable self-management instructions. The median comprehensive PEMAT-P score was 55.60 (54.20–61.10; χ^2^ = 27.01, *P* < 0.001). The EQIP-36 score was highest for DeepSeek-R1 at 39.20 (36.17–44.23) and lowest for Qwen3-Max-Thinking-Preview at 22.20 (20.00–31.00; χ^2^ = 25.07, *P* < 0.001). Regarding readability, all indices showed significant platform differences (Kruskal–Wallis χ^2^ = 10.47–63.50, *P* < 0.001–0.033). DeepSeek-R1 had the lowest FRES of 19.32 (17.94–36.89) and the highest FKGL of 14.28 (13.02–15.85), indicating the greatest reading difficulty. ERNIE Bot 4.5 Turbo and Qwen3-Max-Thinking-Preview had FRES values around 59 [59.34 (53.98–63.00) and 59.25 (53.08–61.10)] and FKGL values around 8 [7.76 (7.18–9.32) and 8.25 (7.72–9.57)], indicating they were easier to read. Overall, these findings suggest that different LLMs balance completeness, actionability, readability, and text length differently, providing a quantitative basis for selecting an LLM for CHF patient education across platforms (see Table 2).

**Table 2:** Characteristics of educational texts on chronic heart failure (CHF) generated by different LLMs.

Variables	Total (*n* = 100)	DeepSeek-R1 (*n* = 20)	Doubao (*n* = 20)	ERNIEBot 4.5 turbo (*n* = 20)	Kimi K2 (*n* = 20)	Qwen3-max-thinking-preview (*n* = 20)	Statistic	*P*-value
Words, M (Q_1_, Q_3_)	285.00 (226.00, 363.00)	530.00 (418.50, 565.50)	290.50 (247.75, 379.50)	306.00 (271.25, 331.75)	241.50 (211.75, 292.50)	201.50 (179.75, 228.75)	χ^2^ = 55.19^#^	**<0.001**
Sentences, M (Q_1_, Q_3_)	21.00 (15.75, 28.00)	37.00 (24.50, 39.50)	23.50 (18.00, 28.25)	25.50 (21.75, 29.00)	14.00 (11.75, 15.00)	16.00 (12.75, 19.00)	χ^2^ = 51.39^#^	**<0.001**
PEMAT-P understandability, M (Q_1_, Q_3_)	69.23 (69.20, 76.90)	76.90 (69.20, 76.90)	69.20 (61.50, 69.20)	69.23 (69.23, 76.92)	76.90 (69.20, 76.90)	69.20 (69.20, 75.00)	χ^2^ = 20.89^#^	**<0.001**
PEMAT-P actionability, M (Q_1_, Q_3_)	20.00 (0.00, 40.00)	26.65 (0.00, 40.00)	0.00 (0.00, 5.00)	30.00 (20.00, 40.00)	20.00 (12.52, 20.00)	20.00 (20.00, 25.00)	χ^2^ = 26.40^#^	**<0.001**
PEMAT-P score, M (Q_1_, Q_3_)	55.60 (54.20, 61.10)	58.95 (55.60, 66.70)	50.00 (50.00, 55.60)	61.10 (55.60, 66.70)	58.35 (55.60, 64.08)	54.20 (54.20, 60.07)	χ^2^ = 27.01^#^	**<0.001**
EQIP-36 score, M (Q_1_, Q_3_)	30.00 (21.65, 38.60)	39.20 (36.17, 44.23)	23.30 (20.00, 30.82)	26.70 (22.20, 34.98)	32.25 (19.30, 40.15)	22.20 (20.00, 31.00)	χ^2^ = 25.07^#^	**<0.001**
FRES, M (Q_1_, Q_3_)	52.81 (39.86, 59.36)	19.32 (17.94, 36.89)	55.48 (53.24, 59.03)	59.34 (53.98, 63.00)	43.60 (38.75, 49.42)	59.25 (53.08, 61.10)	χ^2^ = 63.46^#^	**<0.001**
FKGL, M (Q_1_, Q_3_)	9.63 (8.17, 12.75)	14.28 (13.02, 15.85)	8.58 (8.17, 8.96)	7.76 (7.18, 9.32)	12.65 (10.60, 14.22)	8.25 (7.72, 9.57)	χ^2^ = 63.50^#^	**<0.001**
SMOG, M (Q_1_, Q_3_)	15.00 (14.00, 16.00)	15.00 (14.50, 15.25)	16.00 (15.00, 17.00)	16.00 (15.00, 17.00)	14.00 (12.50, 15.00)	15.00 (14.00, 15.00)	χ^2^ = 28.29^#^	**<0.001**
ARI, M (Q_1_, Q_3_)	13.63 (12.38, 15.14)	15.17 (14.66, 17.19)	13.81 (12.92, 15.26)	13.11 (11.70, 15.57)	13.02 (12.26, 13.75)	13.28 (11.00, 14.03)	χ^2^ = 17.31^#^	**0.002**
CLI, M (Q_1_, Q_3_)	17.01 (14.95, 18.06)	17.46 (14.61, 18.78)	16.61 (14.60, 17.19)	16.38 (13.79, 17.46)	17.94 (17.36, 19.25)	15.98 (14.13, 18.12)	χ^2^ = 14.81^#^	**0.005**
LW, M (Q_1_, Q_3_)	14.07 (11.57, 16.15)	16.30 (14.93, 19.38)	13.30 (11.00, 15.00)	11.85 (10.71, 13.41)	15.43 (14.84, 18.05)	12.40 (11.10, 16.72)	χ^2^ = 29.83^#^	**<0.001**
GFOG, M (Q_1_, Q_3_)	12.83 (11.52, 14.05)	14.03 (12.36, 15.94)	12.68 (11.84, 13.89)	11.58 (10.83, 13.55)	12.89 (12.46, 14.05)	12.18 (11.73, 13.05)	χ^2^ = 10.47^#^	**0.033**
GQS score, M (Q_1_, Q_3_)	5.00 (4.00, 5.00)	4.50 (4.00, 5.00)	5.00 (5.00, 5.00)	5.00 (4.00, 5.00)	5.00 (5.00, 5.00)	5.00 (4.00, 5.00)	χ^2^ = 14.47^#^	**0.006**

# Kruskal–Wallis H test.

M, median, Q_1_, 1st quartile, Q_3_, 3rd quartile. Bold *P*-values indicate statistical significance (*P* < 0.05).

#### Characteristics of chronic heart failure education texts generated by LLMs across different dimensions

In the five dimensions of disease knowledge, daily management and prevention, diagnosis and classification, psychosocial factors and prognosis, and treatment and rehabilitation, there were no significant differences in the length or syntactic complexity of texts generated by LLMs for CHF education. Word count (χ^2^ = 4.93, *P* = 0.295) and sentence count (χ^2^ = 3.30, *P* = 0.508) were similar. Furthermore, there was no significant difference in the PEMAT understandability score (χ^2^ = 7.84, *P* = 0.098). In contrast, the actionability score showed significant variation across the dimensions (χ^2^ = 20.86, *P* < 0.001). The daily management and prevention dimension had the highest actionability [40.00% (29.97–60.00)], which also corresponded to the highest PEMAT overall score [66.70% (58.20–72.20)] and a significant between-dimension difference in PEMAT-P total score (χ^2^ = 16.26, *P* = 0.003). The model was more likely to generate clear actionable advice when the topic included tasks such as weight monitoring, salt and water restriction, symptom alerts, and medication adherence. The disease knowledge dimension had the lowest actionability [10.00% (0.00–20.00)], with the text primarily focused on knowledge dissemination and insufficient guidance for action. Information quality (measured by EQIP-36 score) also showed significant differences (χ^2^ = 22.33, *P* < 0.001), with daily management and prevention scoring the highest [39.70 (25.85–45.32)] and basic understanding of the disease the lowest [21.10 (17.20–30.35)], reflecting different emphases on risk and benefit information, alternatives, uncertainty, and medical indications. Among the readability indicators, FRES (χ^2^ = 6.93, *P* = 0.14) and FKGL (χ^2^ = 5.19, *P* = 0.268) showed no significant differences, while SMOG (χ^2^ = 16.55, *P* = 0.002) and GFOG (χ^2^ = 16.87, *P* = 0.002) differed significantly across dimensions. The treatment and rehabilitation dimension had the highest SMOG score [16.00 (15.00–16.25)], suggesting that related content is more likely to include technical terms and complex expressions, thereby raising reading difficulty for patients. In conclusion, while the model's understandability is relatively stable, actionability and the completeness of information elements are significantly influenced by the topic, indicating that topic selection and task framing may affect the practical utility of LLM-generated CHF education texts (see Table 3).

**Table 3:** Multidimensional characteristics of CHF educational texts generated by LLMs.

Variables	Total (*n* = 100)	Basic understanding of the disease (*n* = 20)	Daily management and prevention (*n* = 20)	Diagnosis and classification (*n* = 20)	Psychosocial factors and prognosis (*n* = 20)	Treatment and rehabilitation (*n* = 20)	Statistic	*P*-value
Words, M (Q_1_, Q_3_)	285.00 (226.00, 363.00)	297.00 (225.25, 332.25)	292.50 (218.00, 427.25)	265.00 (211.75, 300.50)	265.50 (221.00, 363.00)	358.00 (254.25, 458.75)	χ^2^ = 4.93^#^	0.295
Sentences, M (Q_1_, Q_3_)	21.00 (15.75, 28.00)	19.00 (15.00, 23.25)	21.50 (13.75, 29.00)	18.50 (16.00, 23.50)	23.00 (16.00, 29.25)	24.50 (16.50, 36.00)	χ^2^ = 3.30^#^	0.508
PEMAT-P understandability, M (Q_1_, Q_3_)	69.23 (69.20, 76.90)	72.12 (69.20, 76.90)	76.90 (69.20, 76.92)	69.20 (68.58, 71.15)	75.95 (69.20, 76.90)	69.20 (69.20, 71.15)	χ^2^ = 7.84^#^	0.098
PEMAT-P actionability, M (Q_1_, Q_3_)	20.00 (0.00, 40.00)	10.00 (0.00, 20.00)	40.00 (29.97, 60.00)	20.00 (0.00, 20.00)	20.00 (15.00, 20.00)	20.00 (15.00, 23.32)	χ^2^ = 20.86^#^	**<0.001**
PEMAT-P score, M (Q_1_, Q_3_)	55.60 (54.20, 61.10)	55.60 (50.00, 59.00)	66.70 (58.20, 72.20)	54.20 (50.00, 56.70)	58.35 (55.25, 65.73)	55.60 (54.20, 61.10)	χ^2^ = 16.26^#^	**0.003**
EQIP-36 score, M (Q_1_, Q_3_)	30.00 (21.65, 38.60)	21.10 (17.20, 30.35)	39.70 (25.85, 45.32)	23.30 (21.65, 30.25)	30.00 (21.65, 37.60)	34.50 (25.85, 39.40)	χ^2^ = 22.33^#^	**<0.001**
FRES, M (Q_1_, Q_3_)	52.81 (39.86, 59.36)	51.39 (34.16, 56.55)	56.25 (49.12, 60.28)	53.27 (42.52, 60.80)	54.25 (43.55, 60.76)	48.12 (36.28, 54.20)	χ^2^ = 6.93^#^	0.14
FKGL, M (Q_1_, Q_3_)	9.63 (8.17, 12.75)	10.05 (8.58, 13.37)	8.93 (7.76, 10.51)	8.91 (8.07, 13.10)	8.67 (7.77, 12.57)	10.16 (9.01, 13.97)	χ^2^ = 5.19^#^	0.268
SMOG, M (Q_1_, Q_3_)	15.00 (14.00, 16.00)	15.50 (15.00, 16.75)	14.00 (12.00, 15.00)	15.00 (14.00, 16.00)	15.00 (14.50, 16.00)	16.00 (15.00, 16.25)	χ^2^ = 16.55^#^	**0.002**
ARI, M (Q_1_, Q_3_)	13.63 (12.38, 15.14)	14.32 (13.52, 16.97)	13.09 (11.82, 14.83)	12.91 (11.96, 13.87)	13.53 (12.89, 14.51)	13.68 (12.99, 15.08)	χ^2^ = 7.93^#^	0.094
CLI, M (Q_1_, Q_3_)	17.01 (14.95, 18.06)	17.07 (15.99, 19.21)	15.34 (13.97, 17.02)	16.41 (15.16, 17.35)	17.48 (16.14, 18.39)	17.64 (17.09, 18.52)	χ^2^ = 9.14^#^	0.058
LW, M (Q_1_, Q_3_)	14.07 (11.57, 16.15)	15.00 (12.57, 18.05)	13.12 (10.71, 15.51)	13.22 (11.30, 15.50)	13.62 (12.63, 16.62)	15.20 (11.95, 16.62)	χ^2^ = 3.89^#^	0.421
GFOG, M (Q_1_, Q_3_)	12.83 (11.52, 14.05)	13.48 (12.73, 15.17)	12.06 (10.83, 12.88)	11.92 (10.84, 12.76)	12.86 (11.84, 14.31)	13.50 (12.72, 14.05)	χ^2^ = 16.87^#^	**0.002**
GQS score, M (Q_1_, Q_3_)	5.00 (4.00, 5.00)	4.00 (4.00, 5.00)	5.00 (5.00, 5.00)	5.00 (5.00, 5.00)	5.00 (4.00, 5.00)	5.00 (5.00, 5.00)	χ^2^ = 12.46^#^	**0.014**

# Kruskal–Wallis H test.

M, median, Q_1_, 1st quartile, Q_3_, 3rd quartile. Bold *P*-values indicate statistical significance (*P* < 0.05).

### Text quality

**Figure 1:**
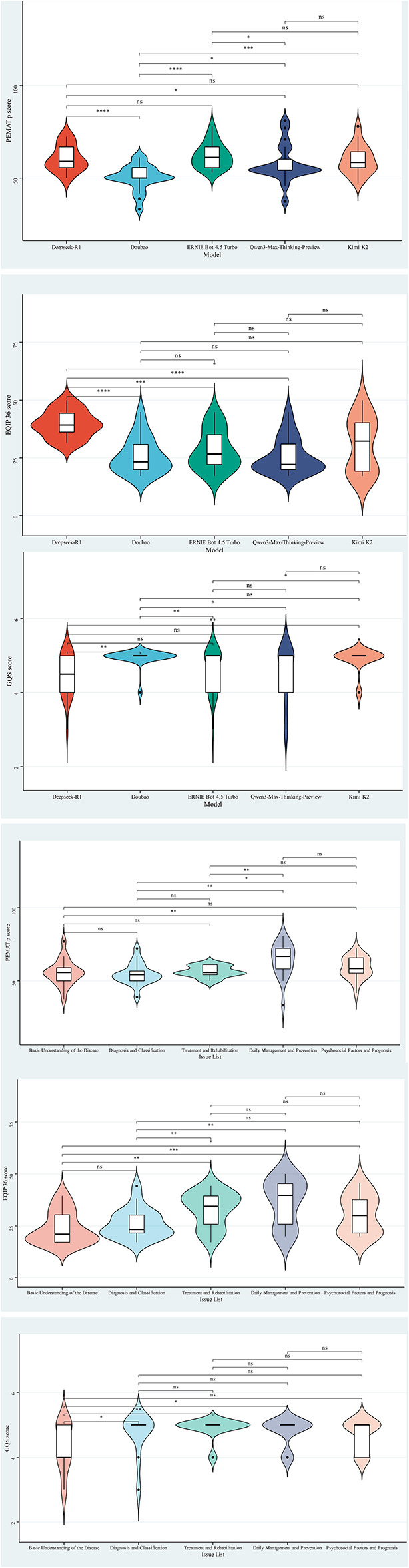
Distribution of actionable, information quality, and overall quality of educational texts on CHF across different platforms and dimensions. *P < 0.05; **P < 0.01; ***P < 0.001; ****P < 0.0001.

Figure 1 compares five platforms on PEMAT-P, EQIP-36, and GQS. Platform differences were significant for all metrics (PEMAT-P χ^2^ = 27.01, *P* < 0.001; EQIP-36 χ^2^ = 25.07, *P* < 0.001; GQS χ^2^ = 14.47, *P* = 0.006). ERNIEBot 4.5 Turbo had the highest PEMAT-P median at 61.10 (IQR 55.60 to 66.70), followed by DeepSeek-R1 and Kimi K2, whereas Doubao was lowest at 50.00 (IQR 50.00 to 55.60), indicating weaker conversion to concrete self-care steps such as monitoring, medication adherence, and when to seek care. DeepSeek-R1 led EQIP-36 at 39.20 (IQR 36.17–44.23); Kimi K2 was lower and more variable at 32.25 (IQR 19.30–40.15). Qwen3-Max-Thinking-Preview and Doubao clustered around 22 to 23, suggesting omissions in risk warnings, evidence support, and completeness. GQS was mostly 4–5, with more five-point ratings for Doubao and Kimi K2, a lower median for DeepSeek-R1 at 4.50, and more low ratings for Qwen3-Max-Thinking-Preview. Before clinical use, platform selection and structured templates with health care professional review may reduce risks in patient education. Quality also differed across five topic domains (Figure 1), with significant topic effects for PEMAT-P (χ^2^ = 16.26, *P* = 0.003), EQIP-36 (χ^2^ = 22.33, *P* < 0.001), and GQS (χ^2^ = 12.46, *P* = 0.014), indicating topic dependence beyond platform differences. Daily management and prevention performed best, with PEMAT-P at 66.70 (IQR 58.20–72.20), actionability at 40.00 (IQR 29.97–60.00), and EQIP-36 at 39.70 (IQR 25.85–45.32), consistent with procedural tasks eliciting stepwise guidance and more complete elements. Foundational disease knowledge scored lowest, with EQIP-36 at 21.10 (IQR 17.20–30.35), GQS at 4.00 (IQR 4.00–5.00), and actionability at 10.00 (IQR 0–20.00), reflecting narrative outputs that often omit risk warnings, clinical thresholds, and uncertainty. Diagnostic and classification and psychosocial and prognostic domains were intermediate, while treatment and rehabilitation scored higher at 34.50 (IQR 25.85–39.40), although general statements still reduced actionability. Structured prompts and health care professional review are recommended for low-actionability topics to improve actionability and completeness and to support self-management.

### Text readability

**Figure 2:**
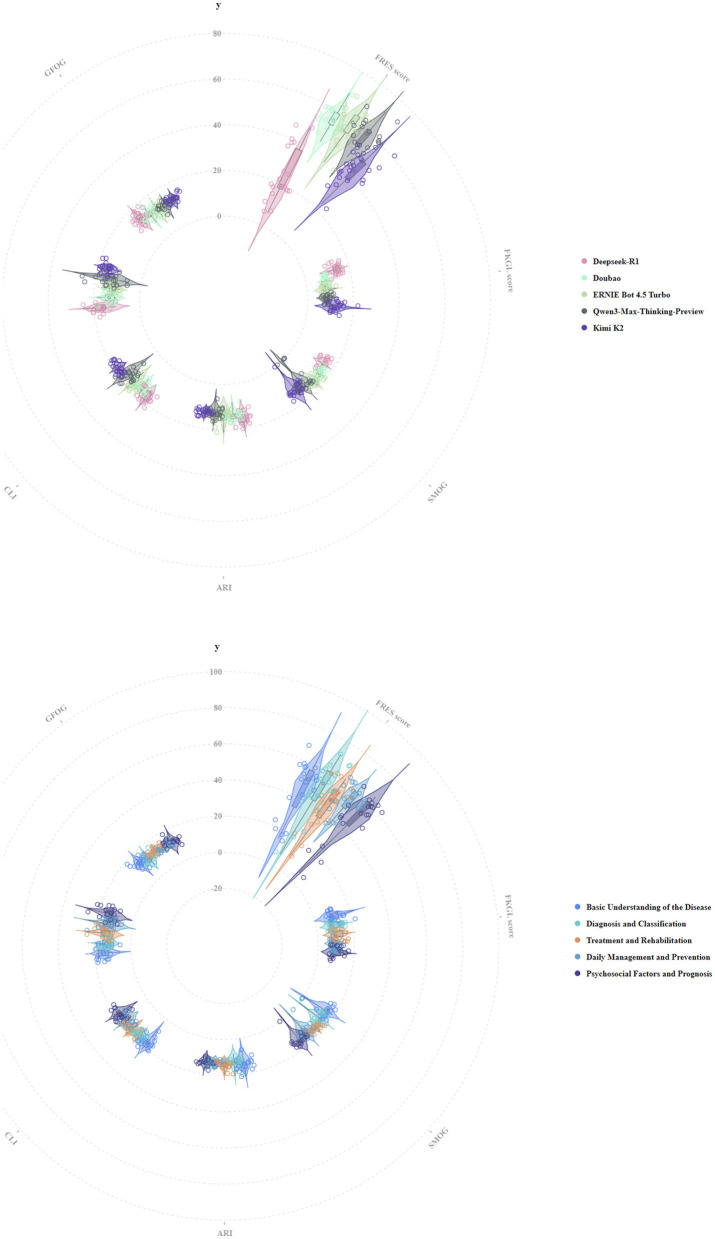
Distribution of readability metrics for educational texts on CHF across different platforms and dimensions.

The polar plot (Figure 2) illustrates the readability stratification of self-management education texts for chronic heart failure generated by different models. The FRES exhibited the highest discrimination. Doubao and ERNIEBot 4.5 Turbo have higher central values and wider distributions, indicating more readable texts, while Qwen3-Max-Thinking-Preview and Kimi K2 are centered in the middle. DeepSeek-R1 demonstrates a lower central value with a larger spread, resulting in more significant variability in readability. The grade-level or complexity indicators, including FKGL, SMOG, ARI, CLI, GFOG, and LW, largely overlap. However, DeepSeek-R1 consistently presents higher central values across several indices, reflecting higher syntactic and lexical complexity. After stratifying by theme, FRES still shows differences: “Diagnosis and Classification” and “Daily Management and Prevention” are more readable, while “Psychosocial Factors and Prognosis” are more difficult and exhibit greater fluctuation. Other complexity indicators show minimal thematic differences.

### Correlation analysis

**Figure 3:**
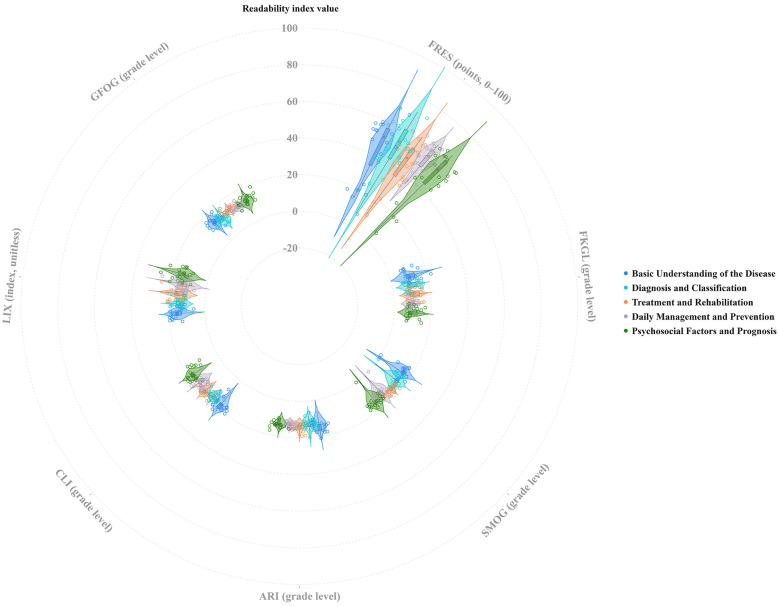
Spearman correlation heatmap of evaluation metrics for LLM-generated CHF self-management education texts pooled across platforms and topics. *P < 0.05; **P < 0.01; ***P < 0.001; ****P < 0.0001.

The correlation heatmaps (Figure 3 in the main text; Supplementary Figures 1–10) showed consistent covariation in text-length metrics within models and within topics. PEMAT-P showed good internal consistency. Actionability had the strongest correlation with the PEMAT-P total score (within-model *r* = 0.56–0.91; within-topic *r* = 0.51–0.88) and was strongly positively correlated with understandability in the daily management and prevention topic (*r* = 0.92). Understandability was generally moderately to strongly correlated with the total score, with substantial between-model variation (within-model *r* = 0.25–0.95). Readability metrics showed a stable clustering pattern: FRES was strongly negatively correlated with FKGL (within-model *r* = −0.81 to −0.98; within-topic *r* = −0.93 to −0.99), whereas FKGL was positively correlated with SMOG, ARI, CLI, and GFOG (approximately *r* = 0.44–0.92). EQIP-36 showed moderate positive correlations with PEMAT-P actionability in some models and topics (approximately *r* = 0.43–0.67). In contrast, GQS was weakly correlated with most metrics and was negatively correlated with word count in the treatment and rehabilitation topic (*r* = −0.49), indicating that readability, information quality, and action-instruction completeness do not necessarily improve in parallel.

After pooling texts across all platforms and topics (Figure 3), Spearman correlations showed three stable patterns. Word count and sentence count were strongly correlated (*r* = 0.82, *P* < 0.001) and were positively correlated with EQIP-36 (word count *r* = 0.48; sentence count *r* = 0.35; both *P* < 0.001), indicating that longer texts tend to include more complete informational elements. PEMAT-P retained good internal consistency: understandability and the total score (*r* = 0.81) and actionability and the total score (*r* = 0.74) were strongly correlated (both *P* < 0.001). Both the total score and actionability were moderately positively correlated with EQIP-36 (*r* = 0.38 and *r* = 0.36, respectively; both *P* < 0.001), suggesting that greater informational completeness is typically accompanied by more executable phrasing. Readability metrics again clustered tightly, with an extremely strong negative correlation between FRES and FKGL (*r* = −0.96, *P* < 0.001). EQIP-36 was negatively correlated with FRES (*r* = −0.29), and word count was negatively correlated with FRES (*r* = −0.38; both *P* < 0.001). Actionability was negatively correlated with SMOG (*r* = −0.28, *P* < 0.01) and LW (*r* = −0.22, *P* < 0.05), suggesting that improved informational completeness may coincide with reduced readability and weaker presentation of actionable key points. GQS remained weakly correlated with most metrics (|*r*| ≤ 0.15), indicating that an overall rating cannot substitute for structured assessments of quality and readability. Overall, the correlation patterns indicate that AI-generated self-management education for chronic heart failure should be optimized concurrently for informational completeness, readability, and checklist-style action steps, rather than prioritizing length or any single metric.

## Discussion

### Key findings

This paper compared and appraised the texts that portray CHF self-management patient education produced by five mainstream Chinese LLMs. The findings displayed substantial variations among the platforms which were mainly reflected in subjective overall quality, objective information completeness and standardization, and readability profiles. It was discovered, based on a combination of GQS, EQIP-36 score, PEMAT-P, and various readability measures, that the conclusions made based on various indicators were not consistent. Platform ranking based on objective quality measures and the subjective overall assessment were distinct, which also implied that one measure is not sufficient to summarize the quality of patient education resources.

Doubao and Kimi K2 showed higher overall quality ratings on GQS, with both platforms achieving a median GQS of 5.00 (5.00–5.00), and significant between-platform differences were observed (Kruskal–Wallis χ^2^ = 14.47, *P* = 0.006). Their productions were more concise and accessible, which may have contributed to higher global ratings. Nonetheless, when assessed using objective quality criteria, both platforms showed gaps in information completeness and verifiability, particularly on EQIP-36 items related to source identification and coverage of key informational elements. DeepSeek-R1, in its turn, had longer outputs [median word count 530.00 (418.50–565.50); Kruskal–Wallis χ^2^ = 55.19, *P* < 0.001], which corresponded to stronger performance in information completeness and standardization. It achieved the highest EQIP-36 score [median 39.20 (36.17–44.23); χ^2^ = 25.07, *P* < 0.001] and a higher PEMAT-P total score [median 58.95 (55.60–66.70); χ^2^ = 27.01, *P* < 0.001]. Nevertheless, it used more technical language and greater informational density, which could increase comprehension burden and lower perceived usability, consistent with its lower GQS [median 4.50 (4.00–5.00)]. Conversely, Qwen3-Max-Thinking-Preview produced shorter outputs [median word count 201.50 (179.75–228.75)] with the lowest EQIP-36 score [median 22.20 (20.00–31.00)], although it was among the most readable, with FRES 59.25 (53.08–61.10) and FKGL 8.25 (7.72–9.57; both Kruskal–Wallis *P* < 0.001). A comparable level of readability was also exhibited by ERNIEBot 4.5 Turbo [FRES 59.34 (53.98–63.00); FKGL 7.76 (7.18–9.32)]. Overall, the pooled correlation patterns supported this trade-off: longer texts tended to include more complete information elements (word count vs. EQIP-36 *r* = 0.48), while readability decreased with increasing length and completeness (FRES vs. FKGL *r* = −0.96; word count vs. FRES *r* = −0.38). This is consistent with the past study on the level of difference in reading among and the level of detail responded by various models studying the same medical problem (35).

These findings indicate that a multi-indicator complementary assessment is more interpretative. The concept of understandability, actionability, medical accuracy, and readability ought to be thought of and a detailed balance of the outcomes need to be taken up. Considering the subjective form of satisfaction as a basis to select the platform in clinical application situation would provide a cloud of information that there may be significant gaps or risks of inaccuracy. However, when the content completeness is considered separately, it can augment the read load and lessen the matter of practical use (36–38). Thus, it is suggested that consistency checks should be performed when selecting the platform, using the content, and in some cases, it is better to add, delete, or even revise the wording to attain a more balanced score between completeness and accessibility of the information.

### Readability and alignment with CHF health literacy assessment

LLMs can help reduce the professional threshold of health-related texts to some extent. However, this study demonstrates that the readability of CHF patient education materials generated by LLMs generally exceeds the level that public health literacy can accommodate. In pooled analyses, the overall FKGL was 9.63 (8.17–12.75), approximating a high-school reading level and remaining above the 5th−6th grade target recommended by the NIH and AMA. Only a few platforms, such as ERNIEBot 4.5 Turbo and Qwen3-Max-Thinking-Preview, produced outputs closer to the middle school level, with median FKGL values of 7.76 (7.18–9.32) and 8.25 (7.72–9.57), respectively. However, these still did not meet the ideal 6th grade threshold. For older adults CHF patients, who generally have low health literacy, such high readability could impair their understanding and implementation of key information. Research has shown that inadequate health literacy among heart failure patients is significantly associated with higher rates of rehospitalization and mortality. Therefore, ensuring that the reading difficulty of educational materials aligns with the patient's cognitive level is crucial to improving the effectiveness of interventions.

Furthermore, advanced LLMs have shown potential in adjusting tone and simplifying expressions. Some studies have demonstrated that by prompting LLMs to rewrite existing health materials, the average reading grade can be reduced from approximately 10th grade to 6th−7th grade, while maintaining content accuracy and understandability (39). This suggests that with more explicit goals for reading levels, terminology replacement, and sentence simplification in prompt engineering, coupled with clinical review and iterative optimization, LLM-generated patient education materials may better align with the needs of populations with varying health literacy, achieving true personalization and improved accessibility.

### Mechanisms behind the insufficient translation from understandability to actionability

This paper established substantial variation in the process of convertibility to actionability among different dimensions of questions. The ability of the five platforms to produce text based on the scale of PEMAT-P revealed the quality of the platform performance that was rather acceptable but little actionable in the ability to generate text. In pooled analyses, the median PEMAT-P understandability score was 69.23% (69.20–76.90; χ^2^ = 20.89, *P* < 0.001), whereas the median actionability score was 20.00% (0.00–40.00; χ^2^ = 26.40, *P* < 0.001). Knowledge questions, including those that concerned disease basic learning and diagnosis and classification, were also very problematic in that the answers to them were not usually concrete and practical in their content. Conversely, questions related to daily management and prevention responses were higher in terms of actionability [median 40.00% (29.97–60.00); χ^2^ = 20.86, *P* < 0.001), probably because of particular actions to undertake–dietary management, weight/symptom monitoring, and lifestyle adaptations.

Regarding the mechanistic approach, LLMs are usually capable of providing answers based on the question alone. The models will have a tendency to give explanatory information, and not behavioral suggestions, when the question ought to seek pathological knowledge or treatment principles. The models do not generally take the initiative of prescribing certain procedures to the patient, negligently leading to low actionability scores, in the absence of any further encouragement. Conversely, even when responding to self-management related questions, some of the suggestions are on the principle level, indicating the missing of a review down of executable steps, and clear action verbs, thus reducing the chances of readers turning the meaning into action.

An assessment of the output of the LLMs in a dental scenario revealed that ChatGPT-4o had relatively high understandability with the PEMAT score surpassing the 70% threshold range, but the actionability of the role played by such models as Claude did not reach the corresponding threshold universally (40). This is an indication that even sophisticated models might still be limited in producing and structuring actionable advice. The detachment of interpretation of information and action can negate the educational benefits of clinically educating patients on self-management behavior, and indirectly on undergoing prognosis due to lack of compliance and other influences. To overcome those problems, design must be prompt and must insist that the model gives specific steps and action tips. The presentation of clear action points, like the oftness of weight monitoring, the quantable standards of the dietary control, and the thresholds in the attendance of medical attention with the red flag symptoms, can also be enhanced by human post-editing, facilitating execution of behaviors basing on knowledge.

### Risks related to medical hallucinations and multi-level governance pathways

Medical hallucinations (fabricated or factually incorrect medical content generated by LLMs) represent one of the core safety risks associated with the use of LLMs for patient education. These models may generate information that appears reasonable but is fundamentally erroneous, potentially leading to misguided decision-making (41). Previous studies have shown that approximately one-third of chatbot-generated disease advice diverges from authoritative guidelines, and around 12.5% contains fabricated treatment recommendations (41). In our evaluation, we noted verifiability concerns, particularly the frequent absence of traceable sources, which can increase the difficulty of review and auditing; however, medical accuracy deviations were not formally audited against contemporary CHF guidelines in this study. Moreover, the variability in uncertainty expression strategies further impacts risk: excessive hedging weakens usability, while overconfidence may amplify the consequences of misinformation. Existing evidence suggests that incorporating information accuracy warnings during the interaction phase can reduce the tendency to generate errors, indicating that engineering solutions may serve as one of the risk mitigation strategies (42).

Therefore, for clinical applications, it is essential to establish a multi-level governance loop that involves collaboration between regulatory bodies, professional organizations, and technology. Regulators and professional groups should integrate generative AI into healthcare quality management, conducting compliance reviews of providers, pre-launch safety evaluations, and targeted scenario-specific audits (43). At the institutional level, content should be subject to review and approval by licensed professionals before publication, following a peer-review-like process. Additionally, multidisciplinary collaboration should be promoted throughout the development and deployment phases (44). From a technological perspective, strategies such as prompts based on authoritative guidelines and evidence tagging, retrieval-enhanced generation to reduce the risk of fabrication, as well as AI self-audits/peer reviews, multi-model cross-validation, and automated audits should be adopted to achieve large-scale verification. Furthermore, enhancing source transparency and traceability is crucial (42–45). Additionally, efforts should be made to improve the AI literacy of healthcare professionals and the public, and to incorporate safety and efficacy validation into ongoing evaluation systems similar to those for drugs and medical devices (41). In conclusion, under clear risk boundaries and governance mechanisms, LLMs can safely realize their potential value in large-scale and personalized patient education.

## Limitations and future perspectives

### Limitations

This cross-platform study provides a standardized comparison of CHF self-management education generated by five mainstream Chinese LLMs; however, several considerations should be noted. First, the evaluation adopted a single time-point, single-prompt, single-turn design. We did not quantify output variability due to stochastic generation, model/version updates, or repeated sampling, and we did not assess the potential benefits of multi-turn conversational refinement; therefore, findings primarily reflect performance under the tested settings and time window. Second, the task set focused on typical CHF self-management scenarios and did not cover broader clinical complexity, which may influence real-world applicability. Third, although standardized English prompts improved comparability across platforms, this design choice may differ from routine Chinese-language patient–clinician interactions, and caution is warranted when generalizing to Chinese-only communication contexts. Finally, our framework emphasized text quality, readability, and actionability; we did not conduct a systematic guideline-based medical accuracy (guideline concordance) assessment or formal verification of cited evidence. In addition, the study evaluated text-only outputs without direct testing of patient comprehension, behavioral adherence, or clinical outcomes, which should be established through prospective user-centered studies.

### Future perspectives

Future research can extend this work in four directions. First, multi-timepoint and repeated-sampling designs can quantify within-model variability and performance stability across model/version updates, and multi-turn conversation tasks can better represent patient-education workflows involving clarification, personalization, and iterative refinement. Second, a guideline-based framework for medical accuracy and safety should be added by mapping generated recommendations to contemporary CHF guideline domains, including medication use, diet and fluid management, exercise, symptom monitoring, and escalation for warning signs, and by scoring guideline concordance with structured checklists or rubrics; independent clinician reviewers and inter-rater reliability should be reported. Third, prospective user-centered studies should evaluate effects beyond text metrics, including patient comprehension, perceived usefulness, self-efficacy, adherence behaviors, and clinically relevant outcomes such as symptom recognition and timely care-seeking; stratified analyses by health literacy may help identify differential benefit and guide content tailoring. Fourth, implementation strategies may integrate retrieval-augmented generation, evidence-linked prompting, standardized output templates, and post-generation safety checks, combined with clinician audit and ongoing monitoring, to improve traceability and mitigate misinformation risk. Collectively, these approaches can enhance the safety, auditability, and real-world applicability of LLM-based CHF patient education.

## Conclusion

This paper critically appraised the CHF self-management education writings produced by DeepSeek-R1, Doubao, ERNIEBot 4.5 Turbo, Qwen3-Max-Thinking-Preview, and Kimi K2 in response to standard prompts and standard questions. Findings showed that overall quality was high, with a median GQS of 5.00 (4.00–5.00), while significant between-platform differences were observed (Kruskal–Wallis χ^2^ = 14.47, *P* = 0.006). Nevertheless, there were marked disparities among platforms, reflecting a structural trade-off between informational completeness/standardization and readability. Doubao and Kimi K2 had the best subjective overall ratings on quality [both with a median GQS of 5.00 (5.00–5.00)]. DeepSeek-R1 achieved the highest information completeness and standardization on EQIP-36 [median 39.20 (36.17–44.23); χ^2^ = 25.07, *P* < 0.001], yet its readability was the lowest, with FRES 19.32 (17.94–36.89) and FKGL 14.28 (13.02–15.85; both *P* < 0.001). Qwen3-Max-Thinking-Preview and ERNIEBot 4.5 Turbo scored higher in readability with FRES 59.25 (53.08–61.10) and 59.34 (53.98–63.00), and FKGL 8.25 (7.72–9.57) and 7.76 (7.18–9.32), respectively (both *P* < 0.001), but their information completeness was lower, reflected by lower EQIP-36 scores [median 22.20 (20.00–31.00) and 26.70 (22.20–34.98), respectively].

There was an understandability–actionability gap in the generated texts as indicated by the PEMAT-P results: overall actionability was low [median 20.00% (0.00–40.00); χ^2^ = 26.40, *P* < 0.001], despite moderate understandability [median 69.23% (69.20–76.90); χ^2^ = 20.89, *P* < 0.001]. This gap was more apparent in knowledge-based areas (e.g., disease basics and diagnosis/classification), whereas actionability was higher for daily management and prevention [median 40.00% (29.97–60.00); χ^2^ = 20.86, *P* < 0.001].

Finally, there are considerable scalable and customizable opportunities in CHF patient education using LLMs across platforms. Nevertheless, existing outputs require refinement in terms of readability alignment, actionability, and source verifiability, alongside strengthened patient safety safeguards. Future work should combine search-enhanced and structured generation techniques and develop a governance loop linking prompt engineering, clinician co-review, and continuous monitoring to minimize the risk of medical hallucinations and improve the usability and real-world impact of patient education.

## Data Availability

The original contributions presented in the study are included in the article/Supplementary material, further inquiries can be directed to the corresponding authors.
