# Supplementary material for: Challenges of using generative AI for patient education in chronic heart failure: an evaluation of content quality, readability, and actionability in cross-platform LLM-generated texts
Source: Front Public Health. 2026 Mar 5;14:1801829. doi: 10.3389/fpubh.2026.1801829 (PMC12999856; doi:10.3389/fpubh.2026.1801829)
Supplement: Supplementary file 1 [file Table_1.docx]

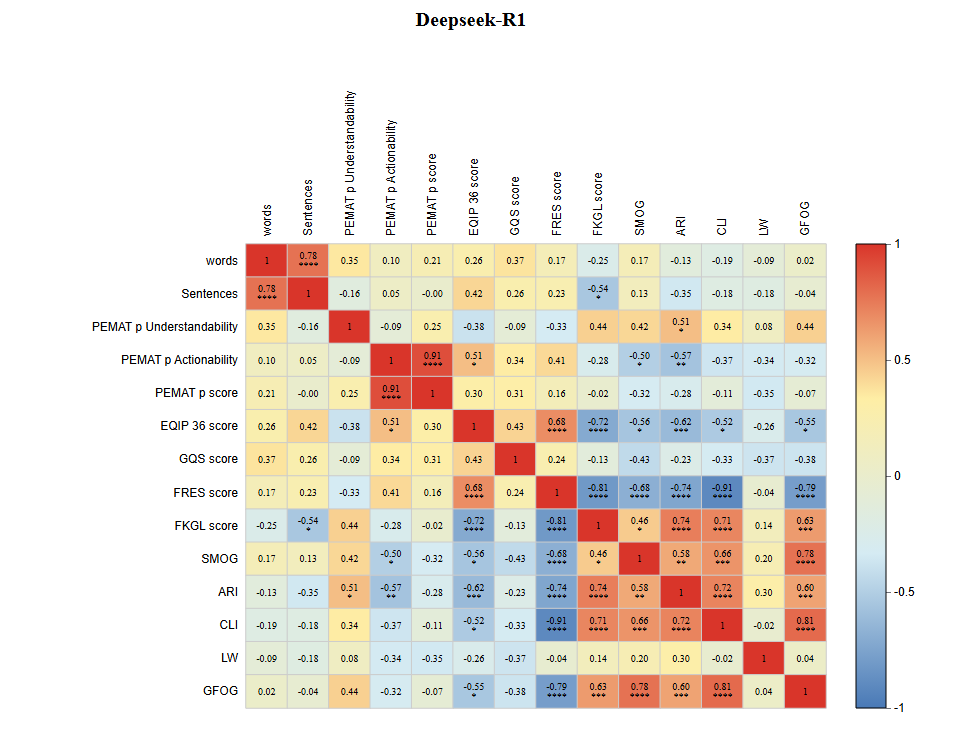


Supplementary Figure 1. Model-specific correlation heatmap: DeepSeek-R1


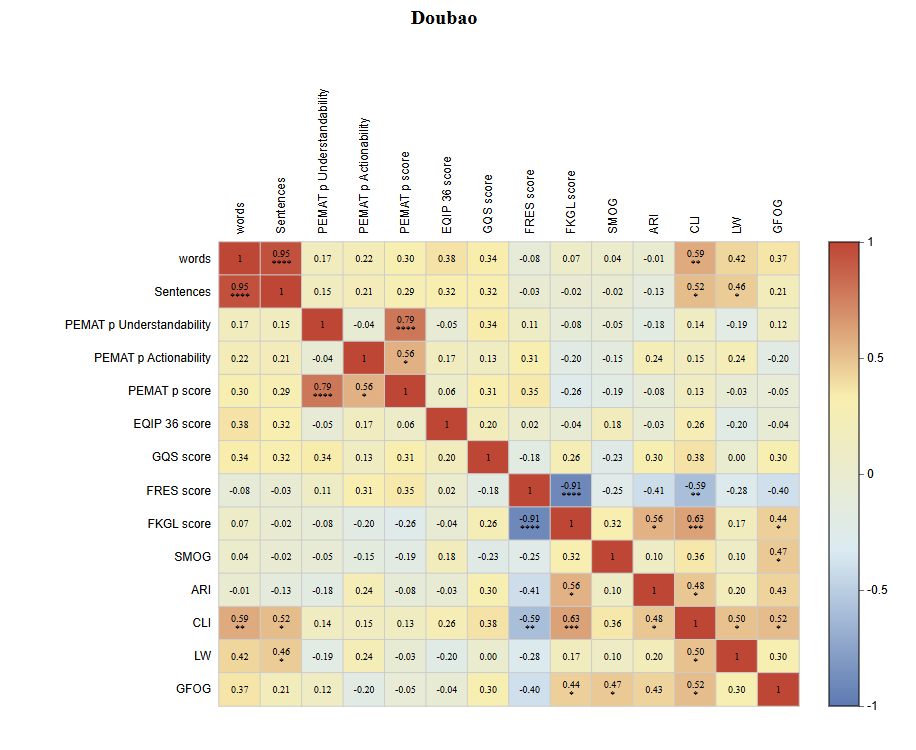


Supplementary Figure 2. Model-specific correlation heatmap: Doubao


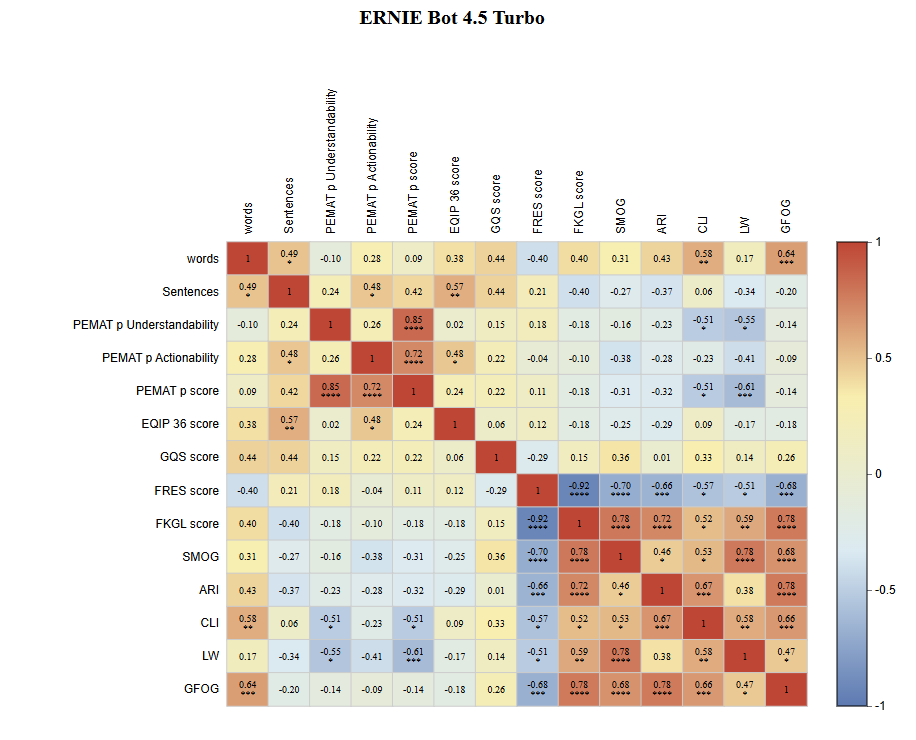


Supplementary Figure 3. Model-specific correlation heatmap: ERNIE Bot 4.5 Turbo


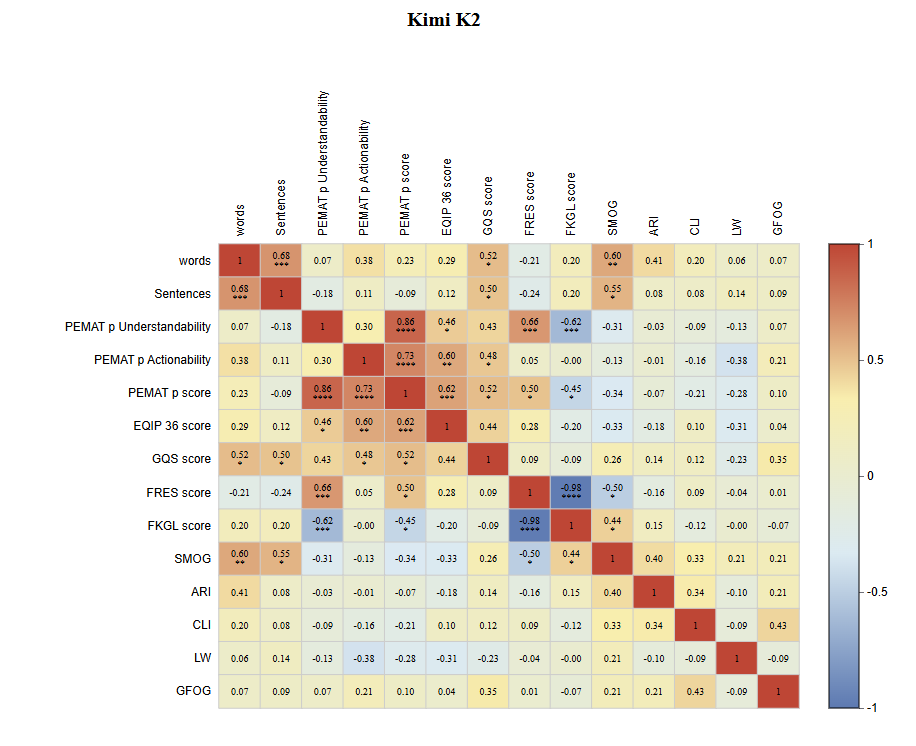


Supplementary Figure 4. Model-specific correlation heatmap: Kimi K2


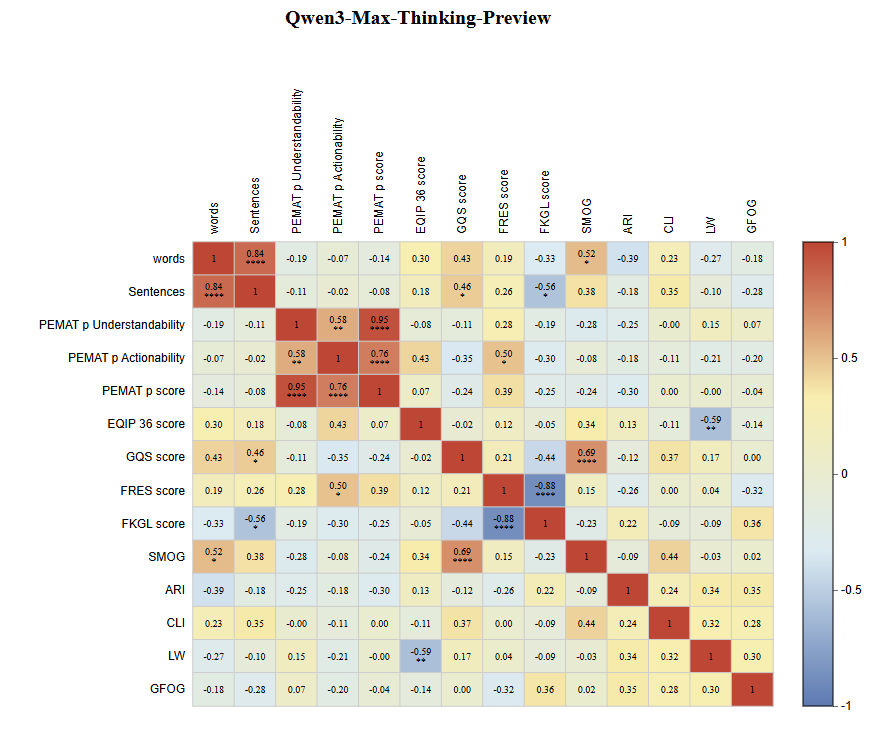


Supplementary Figure 5. Model-specific correlation heatmap: Qwen3-Max-Thinking-Preview


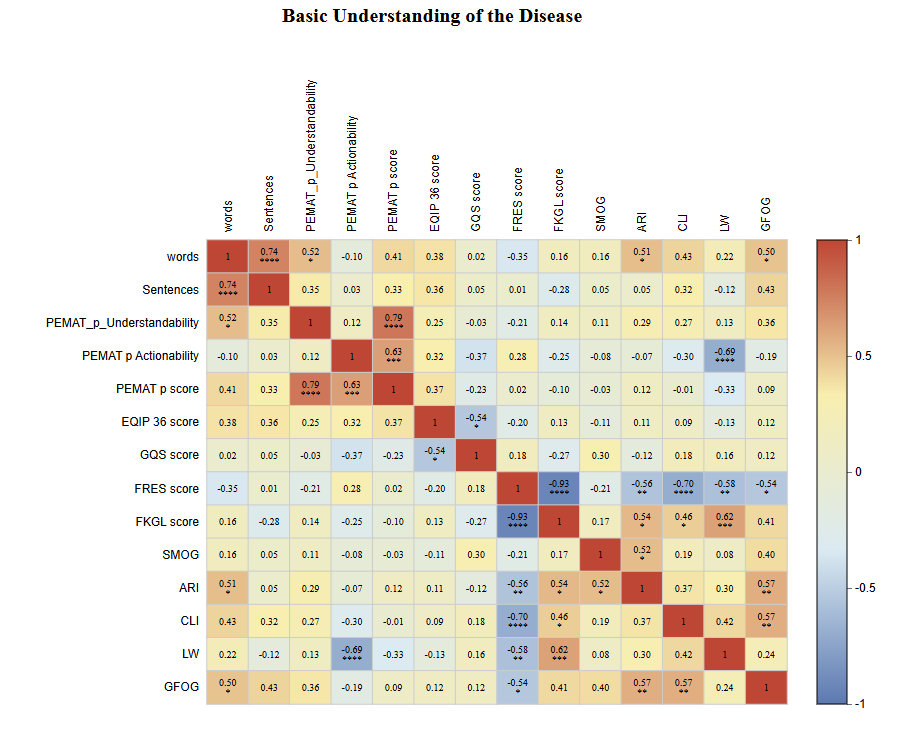


Supplementary Figure 6. Topic-specific correlation heatmap: Basic understanding of the disease


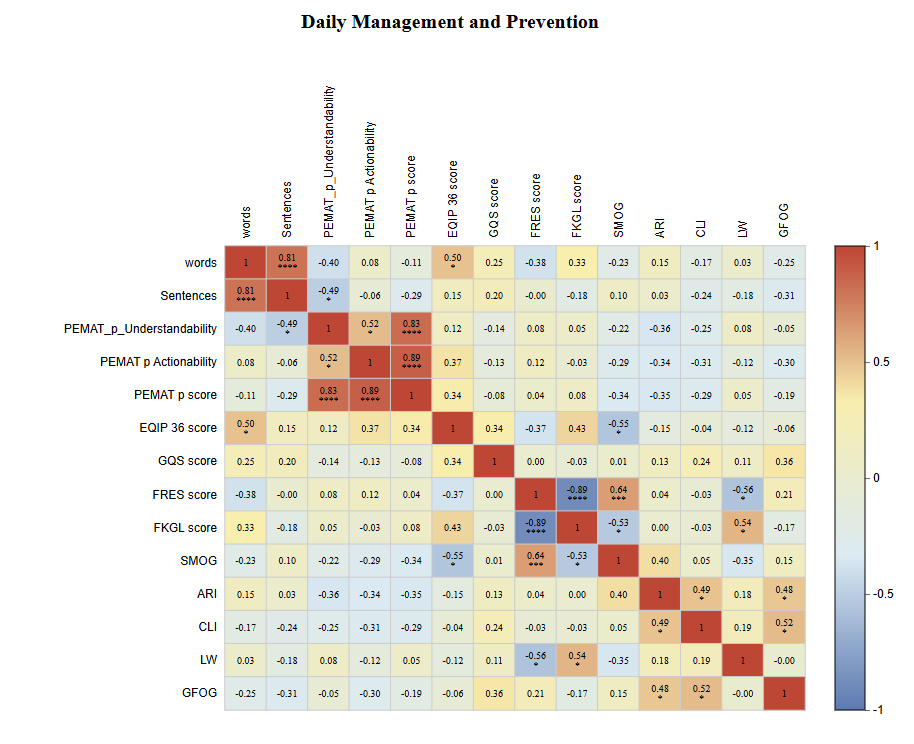


Supplementary Figure 7. Topic-specific correlation heatmap: Daily management and prevention


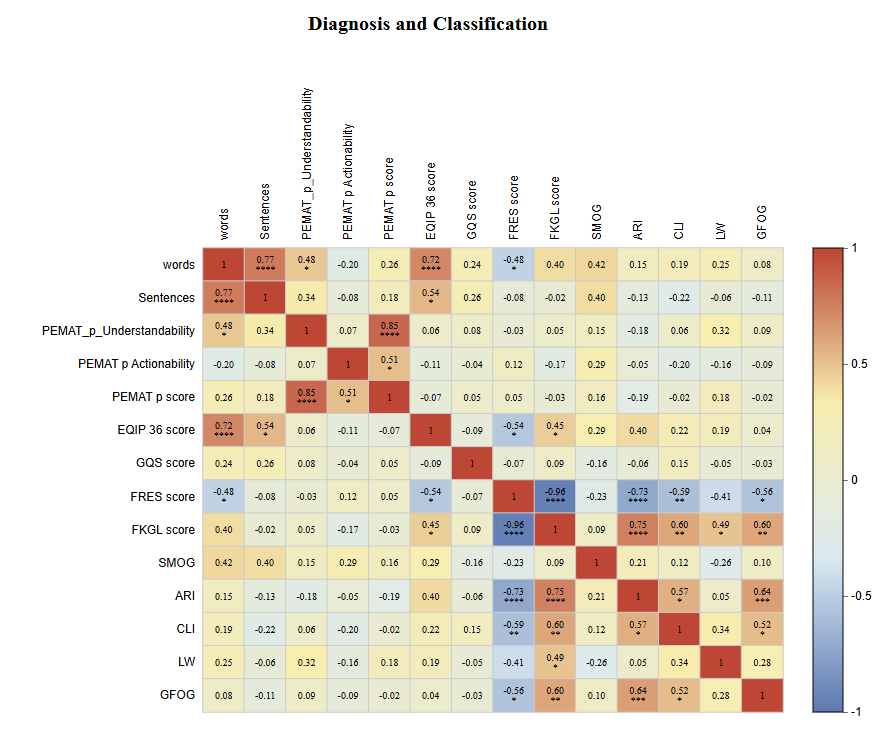


Supplementary Figure 8. Topic-specific correlation heatmap: Diagnosis and classification


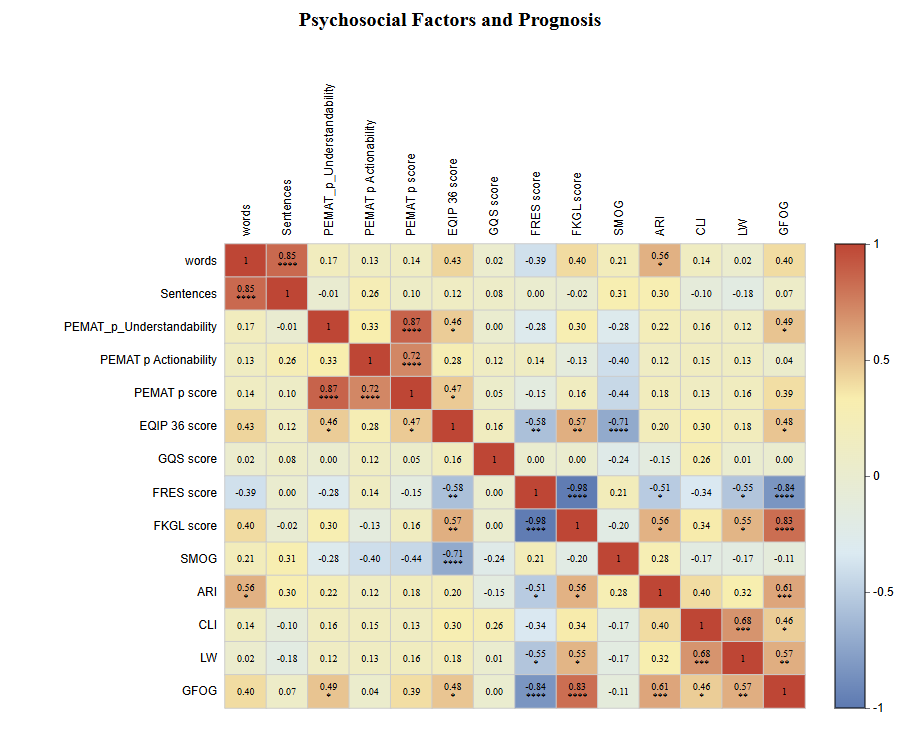


Supplementary Figure 9. Topic-specific correlation heatmap: Psychosocial factors and prognosis


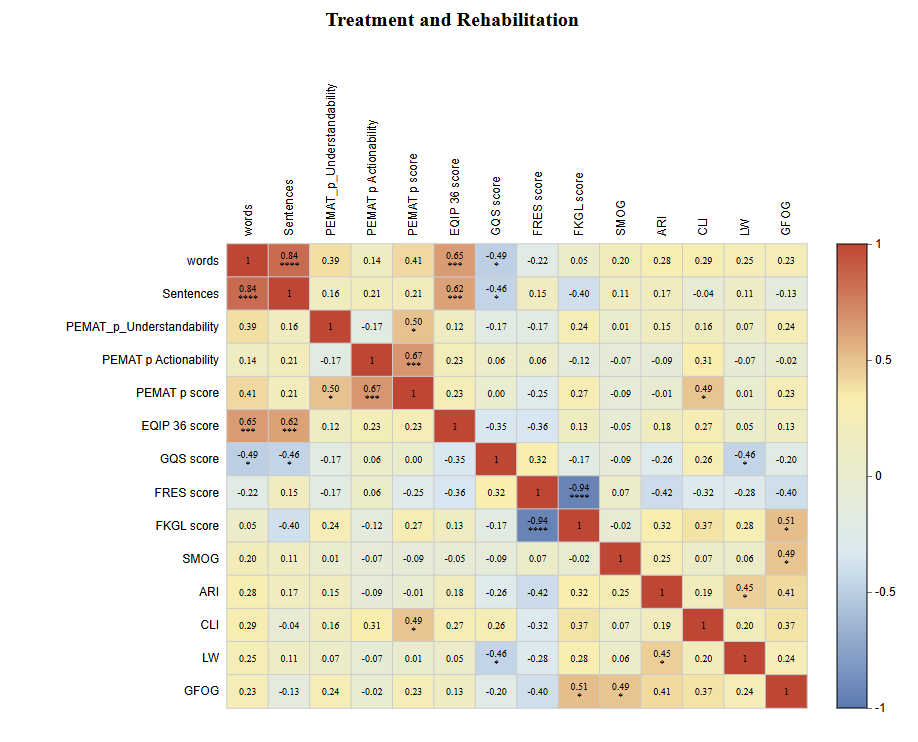


Supplementary Figure 10. Topic-specific correlation heatmap: Treatment and rehabilitation
